# The Institutional Worker

**Published:** 1920-03-13

**Authors:** 


THE
### INSTITUTIONAL WORKER
**Being a Special Supplement to "The Hospital"**

### OUR BUREAU OF INFORMATION.

### Rules for Correspondents.

1. Every letter must be accompanied by the coupon to be cut from the back cover (inside page) of The Hospital, current issue, and must contain the name and address of the correspondent with pseudonym for publication if desired. Replies by post cannot be given save under exceptional circumstances at the Editor's discretion.
2. Letters from Approved Homes in reply to special needs published in the Bureau should state terms and full particulars, and be sent prepaid under cover to the Editor of the Bureau with name written across coupon for identification.
3. Proprietors of Homes which have not yet been entered on the List of Approved Homes, but have spare accommodation likely to suit special needs, are invited to write for an application form for registration. The fee for registration, which includes two announcements of the Home in the Bureau and other privileges, is 10s.
4. All communications to be addressed to the Editor of The Hospital, 28 Southampton Street, Strand, London, W.C. 2, and marked "Bureau of Information."

### INSTITUTION FACTS AND FIGURES.

### IN THE SURGERY OF AN IRONWORKS.

There will always be a certain number of nurses to whom neither private nor institutional nursing will appeal very strongly. They prefer a more "independent" line, where they can live in their own rooms and have fixed hours for their work. The *rôle* of school nurse, under various educational authorities, has, of course, long provided one such opening, and another is increasingly to be found in the position of the nurse, or "Matron," which is being created in many large works and factories.

Some months ago the managing director of a large ironworks in the Black Country heard that I was interested in the subject, and kindly arranged for me to meet the nurse who had been working for some time among his own employees, some 2,000 in number. A small boy was deputed to take me to "Matron," and we crossed the stone-paved yard, seamed with railway lines and noisy with the clang and crash of machinery from surrounding foundries and shops. At a small door marked "Surgery" and exhibiting the times when Matron could be seen, we stopped and she herself welcomed me in. The room was divided into two. The outer and much larger compartment contained a couch, a white-tiled sink with a lighted geyser beside it, a table with drawers of rolled bandages, lint, etc., and a shelf upon which stood a row of bottles bearing familiar labels, Boracic, Sal Volatile, Lysol, etc. The inner room was very tiny, nurse's own sanctum, in fact, but used occasionally for one patient to wait in while a second was having attention in the surgery proper. A small fire burned in the grate and before it stood two basket-work chairs; on the distempered walls were several framed photographs, and under the window stood a desk with writing materials and a beautifully-kept "Case Book." "Date,", "Complaint," "Remarks," headed the three columns into which each page was divided, and under the second I read, "Crushed leg, torn finger, cinder in eye, burnt foot."

"Do you principally get things like these," I asked, "minor accidents?" "Principally," nurse answered, "but, of course, we can have very bad ones sometimes. Not only fractures, but very severe hæmorrhage. I do what I can here while they are sending for the ambulance, and if it is a bad case I go with it to the hospital myself."

"Do you visit in the homes at all?" I asked. Nurse shook her head. "Not to give treatment," she answered. "If it is a comparatively small thing needing just a simple dressing perhaps, the patient comes to me here each day and I attend to it. Otherwise I refer them to their Panel doctors. The only cases I visit are those in which some special help has been asked for, on account of illness, and the directors ask me to visit and report to them."

"It has been suggested that ex-V.A.D.s might take this kind of post," I said; "what do you think about that?" Nurse considered.

"Judging from my own experience I should say 'No,'" she answered. "You see it is not only accidents that one has to deal with. People come for advice about all sorts of things, so that one really has to try and diagnose very often." She turned the pages of her Case Book. "Indigestion, neuritis, impetigo, toothache," she read out. "They come with all sorts of aches and pains, so that a V.A.D. would need to be exceptionally experienced and well trained." "And not very young?" I suggested.

"No, certainly not very young," nurse agreed, "there are a great many married women employed, and part of my work is to keep an eye on them. When one is nearing her confinement I say a word to the foreman, and she is often put on a lighter or more suitable job. But what makes it difficult for me is that they are so afraid of losing their employment they constantly deny that they are pregnant when I know that they are. If an unmarried girl is about to become a mother I find that if I say to her, 'Tell your young man that Matron has spoken to you, and you will have to leave soon, and perhaps he will marry you,' the man very often does so before the baby arrives. Otherwise I try to get the girl into a home in the town of which the superintendent has always been very kind in helping me." "It is not easy work," I said, "it must need great tact and patience." "No, it is not easy work," nurse admitted, "and often it is very disappointing. Of course we have a very rough type of worker here—the roughest and most ignorant I have ever had to deal with—and the work naturally varies with the class of the employees. When I came first they did not want me at all, but they have just asked the directors to appoint a second nurse, so they have evidently got over their suspicions."

She laughed, but I reflected that she had indeed made good.

"Do you need a second nurse?" I asked.

"We could, of course, do more actual welfare work," she answered, "I can only do what I have time for apart from my other work. In that I have a very good assistant in one of the mess-room girls. We had some first-aid lectures here in the winter, and she was particularly keen. I coached her for the examination, and now I can leave her in the surgery if I am called to a distant part of the works, as she is quite capable of dealing with minor cases herself."

There was a knock at the door and a lad entered shading his eye with his hand. I said "Good-bye" to nurse, and as I crossed the noisy yard I felt that it was indeed difficult work, but at the same time work well worth the doing.—H. M. N.

### Question for March.

### WIDENING THE AREA OF HOSPITAL SUPPORTERS.

Elaborate a plan for obtaining the financial assistance of members of the middle classes who do not at present contribute to hospital funds and who cannot afford to become annual subscribers. Outline methods of working, without involving an increase of staff.

**(A minimum payment of 10s. 6d. will be made for each published answer.)**

### RULES.

The following rules must be observed :—

1. Contributions must be written on one side of the paper. Conciseness and terseness are desirable features. The MS. must bear the name and address of the sender and be accompanied by coupon to be cut from the back cover (inside page) of the current issue of The Hospital. A pseudonym must be chosen if the name is not to be published.

2. Contributions must be addressed to the Editor of The Hospital Institutional Worker Supplement, 28 & 29 Southampton Street, Strand, London, W.C. 2, should reach him before the end of the current month, and be marked in the left-hand corner "Facts and Figures."

### QUESTIONS INVITED.

In connection with our Question Box, we offer every month five shillings each for the best questions which are sent in for consideration. The questions may be on any institutional subject and concern any department, from the secretary's to the porter's, or the matron's to the domestic staff; the one essential is that they must be practical and deal with points that have a definite relation to hospital or institutional administration, upkeep, finance, or management. Points relating to artificers' work, housekeeping, the laundry, out-patients, and other practical matters will be welcome. It is hoped that thus, when difficult or doubtful points arise in the course of their work, institutional workers will be encouraged to put them in the form of a question and send them to the Editor, The Hospital Bureau of Information, 28 & 29 Southampton Street, Strand, London, W.C. 2, marked "Question Box."

The best questions will be published in due course, and our readers will be given the opportunity of answering them. Thus all institutional workers may be able to cooperate with the view of helping the work and smoothing the difficulties of each other. In short, we want the Question Box of the *Institutional Worker* to be freely used by every worker habitually.

### ENQUIRIES AND ANSWERS.

### *EMPLOYMENT AND TRAINING.*

### Prospects of a Masseuse.

It is somewhat difficult to advise you upon the question of taking up massage as a profession. The demand for trained masseuses at the present time is certainly great, but unfortunately for many the supply far exceeds the demand. If you wish to do private work only, unless you have friends in the medical profession who can guarantee you work when you have qualified, you will find it is very uphill work to get a big connection. On the other hand, if you prefer work in an institution, we should suggest that you obtain your S.R.E. certificate as well as that for massage and medical electricity. The whole course would extend over fifteen months.—Anxious.

### Training in Chiropody.

The only chiropody training-school we know of is that organised by the Incorporated Society of Chiropodists, 1 Silver Street, Bloomsbury, W.C. 1. Write to the Secretary for particulars of training. You will find "First Aid for Foot Troubles," by Ernest G. L. Runting, and published by The Scientific Press, Ltd., 28 & 29 Southampton Street, Strand, W.C. 2, very helpful.—Agnes.

### C.M B Examinations.

New regulations in regard to the dates at which examinations under the Central Midwives Board will be held have been drawn up recently. For the future examinations will be held every two months in London and every two months alternately in Birmingham and Bristol, in Manchester and Liverpool, and in Leeds and Newcastle-on-Tyne. The address of the Central Midwives' Board is Caxton House, Tothill Street, Westminster, S.W.—Enquirer.

### Recognised Training-Schools.

We give you below a few recognised training-schools, but space does not permit of our giving you the full list you ask for. You will find a full and comprehensive list in "How to Become a Nurse," published by The Scientific Press, Ltd., 28 and 29 Southampton Street, Strand, W.C. 2, price 2s. 10d. post free. Derbyshire Royal Infirmary; Chesterfield and North Derbyshire Hospital; Coventry and Warwickshire Hospital; Royal Devon and Exeter Hospital, Exeter; and Royal Surrey County Hospital, Guildford.—Kate.

### Training for Health Worker.

Write to the Secretary of the Royal Sanitary Institute, 90 Buckingham Palace Road, London, S.W., for a syllabus of lectures and demonstrations for the spring term. The term commenced during the first week in February, so it would be advisable for you to inquire whether it is too late for you to enter this session.—B. R.

### Examination of Medico-Psychological Association.

If you have already obtained a year's training in a general hospital you will be exempt from the preliminary examination for the Medico-Psychological certificate. You will be eligible for the final examination after training for a further period of two years in not more than two institutions for the insane. Write to the Secretary of the Association at 11 Chandos Street, London, W., for any further particulars.—Nurse.

### *MISCELLANEOUS.*

### Nurses' Club in Edinburgh.

The address of the new Nurses' Club in Edinburgh is 8 Drumsheugh Gardens. Membership is open to nurses trained in general, fever, mental, or midwifery nursing, and to probationers who have been in training for not less than six months. Members of the Edinburgh Centre of the College of Nursing pay 7s. 6d. per annum, and members of other centres 5s. for six months. Trained nurses who are not members of the College pay 10s. 6d. per annum and 7s. 6d. for six months. A bed can be obtained for 2s. 6d. for one night by members, and 3s. for non-members. Breakfast, lunch, tea, and dinner can be obtained at reasonable charges. Application should be made to the Lady Superintendent.—Jean.

### Local Centre of College of Nursing in Leicester.

Yes, there is a local Centre of the College of Nursing, Ltd., in Leicester. Apply for membership of the Local Centre to the Hon. Secretary, Miss Masters, Poor-Law Infirmary, Leicester.—College Member.

### New Electrical Apparatus.

The new "Physio" Faradic Wave apparatus which you refer to can be obtained of the Medical Supply Association, Ltd., 167-185 Gray's Inn Road, London, W.C. 1. It is maintained that the current derived from this apparatus is painless, and contractions can be obtained by it at the rate of from sixty to 3,000 per minute. A metronome interrupter is not necessary for use with this apparatus, as it is fitted with an electro-mechanical pendulum beam wave governor, which provides a greater range of interruptions than the former. The price is £7 17s. 6d., net complete.—Medical Electrician.

### Interesting Pamphlet.

Some suggestions as to the duty of the State in the control of venereal disease has been drawn up by a Committee of the Medical Women's Federation and approved by the members at a general meeting. These suggestions have been published in the form of a pamphlet, and can be obtained of the Medical Women's Federation, 9 Clifford Street, New Bond Street, London, W. 1.—K. S.

